# Penetrating Vascular Injuries of the Lower Limbs after Stab Wounds: Predictive Factors of Limb Loss and Mortality

**DOI:** 10.3390/jcm12103476

**Published:** 2023-05-15

**Authors:** Bilel Derbel, Daniela Mazzaccaro, Nidhal Krarti, Rim Miri, Yassine Khadhar, Melek Ben Mrad, Paolo Righini, Giovanni Nano, Raouf Denguir

**Affiliations:** 1 Faculty of Medicine of Tunis, University of Tunis El Manar, Tunis 1002, Tunisia; 2 Cardiovascular Surgery Department, La Rabta Hospital, Tunis 1007, Tunisia; 3 Operative Unit of Vascular Surgery, IRCCS Policlinico San Donato, San Donato Milanese, 20097 Milan, Italy; 4 Department of Biomedical Sciences for Health, University of Milan, 20161 Milan, Italy

**Keywords:** stab wounds, lower extremity, predictive factors, amputation, mortality

## Abstract

Background: Penetrating vascular injuries (PVIs) of the lower limbs due to stab wounds are associated with high mortality and limb loss rates. We analyzed the outcomes of a series of patients who underwent surgical treatment of these lesions, assessing the presence of any factor associated with limb loss and mortality; (2) Methods: Data of patients admitted from 01/2008 to 12/2018 were retrospectively analyzed. Primary outcomes were the limb loss and the mortality rate at 30 days postoperatively. Univariate and multivariate analyses were performed as appropriate. *p* values < 0.05 were considered significant; (3) Results: Data of 67 male patients were analyzed. Two died (3%) and three (4.5%) had a lower limb amputation after failed revascularization. In the univariate analysis, the clinical presentation significantly affected the risk of postoperative mortality and limb loss. The location of the lesion at the superficial femoral artery (OR 4.32, *p* = 0.001) or at the popliteal artery (OR 4.89, *p* = 0.0015) also increased the risk. In the multivariate analysis, the need for a vein graft bypass was the only significant predictor of limb loss and mortality (OR 4.58, *p* < 0.0001); (4) Conclusions: PVIs of lower limbs due to stab wounds were lethal in 3% of cases and lead to a secondary major amputation in 4.5% more cases. The need for a vein bypass grafting was the strongest predictor of postoperative limb loss and mortality.

## 1. Introduction

Penetrating vascular injuries (PVI) of the lower limbs due to stab wounds are associated with a high risk of limb loss and mortality [1]. These types of injuries, once seen mostly in the war zones [2], are nowadays increasing among civilians due to the increase of violence in our society because of drug abuse and alcoholism. In addition, more recent wars such as in Ukraine, Iraq, and Afghanistan may have further increased the incidence of such lesions.

Surgical management of PVI has evolved historically, from vessel ligation with consequent high rates of amputations experienced during early military reports, to the attempt of early vascular repair during the Vietnam and Korean wars with superior limb salvage rates [3,4,5]. 

The improvement in surgical techniques and the more expeditious referral to the appropriate vascular centers have both played a key role in decreasing the mortality and amputation rates, but the management of these injuries in civilians still remains challenging, and the optimal strategies are still under investigation [6]. 

The aim of our study was to analyze retrospectively the outcomes of a cohort of patients who underwent surgical treatment of PVI of the lower limbs due to stab wounds in an urban level 1 trauma center, assessing the presence of any factor associated with limb loss and mortality.

## 2. Materials and Methods

Local Ethics Committee approval was obtained for this retrospective study. Data of patients consecutively admitted to our department for a PVI of the lower limbs (arterial and/or venous) due to stab wounds from January 2008 to December 2018 were retrospectively collected and analyzed. 

Patients presenting with abdominal or thoracic associated injuries were excluded from the analysis, as well as patients with iatrogenic (interventional, orthopedic) or gunshot vascular injuries. 

Data were retrospectively collected based on information taken from the patients’ files and the operative reports by a single investigator, and recorded in a collection form. The data collected included patients’ demographics (age, sex), initial clinical presentation (with particular attention to hemodynamic status and presence of either hard or soft signs of vascular injuries), the presence or absence of local hemostatic compression on admission, the mechanism of injury, the site of injury, the presence of any associated injuries, type of injury on surgical exploration and the type of surgical procedure performed.

Laboratory data included hemoglobin level and the need for transfusion. The results of specific diagnostic studies including duplex ultrasonography and computed tomographic angiography (Angio-CT) also were collected and correlated with physical examination. 

During postoperative course, the occurrence of any complications and the length of stay were recorded.

### 2.1. Definitions 

A state of hemorrhagic shock was defined by a systolic measured blood pressure under 80 mmHg, while a state of circulatory collapse was defined by systolic measured blood pressure ranging between 80 and 99 mmHg.

The presence of hard signs of vascular compromise were defined as pulsatile bleeding, expanding or pulsatile hematoma, presence of a bruit or palpable thrill, absent or diminished pulses or signs of distal ischemia as demonstrated by the presence of pain, pallor, paresthesia, nerve paralysis and poikilothermia.

Patients presenting with hard signs of vascular injury were then sent to the operatory room for immediate surgical exploration after a first level, bed-side imaging, such as an echo-Duplex scan. In patients with soft signs of vascular injury (non-pulsatile hematoma) with a persistent pulse and a warranted hemodynamic stability, additional diagnostic imaging was requested at the discretion of the attending vascular surgeon [7].

### 2.2. Surgical Management 

Standard open surgical techniques for revascularization were used to repair venous and arterial injuries, such as graft bypass or interposition using autogenous great saphenous vein or prosthetic graft, vascular reconstruction using direct end to end anastomosis, direct arteriorrhaphy/venorrhaphy and vascular ligation. As a rule, vascular injuries were repaired before any of the associated injuries. For nerve injuries, plastic surgeons were solicited for evaluation and repair. 

Patients presenting with a tense compartment syndrome received a calf fasciotomy through both medially and laterally placed incisions. 

Failure to restore direct arterial flow to the ankle pulsation along with the persistence of signs of limb ischemia indicated a procedure failure. In these cases, the general status of the patient, the operation performed and the findings were re-evaluated and a decision was made regarding re-exploration or conservation. 

Then, amputation was carried out whenever limb salvage was deemed to be impossible, such as for extremely mangled limbs, or when an extended gangrene and nonviable superficial posterior compartment in addition to either an anterior or lateral compartment were present. 

### 2.3. Statistical Analysis

The statistical analysis was performed using SPSS^®^ 23 software (IBM SPSS, Turkey). Categorical variables are presented as proportions and continuous variables as mean + standard deviation or median (interquartile range [IQR], minimum-maximum) as appropriate.

The primary outcomes were the limb loss rate and the mortality rate at thirty days postoperatively.

Univariate and multivariate analysis with logistic regression and one-way ANOVA tests were used to evaluate the presence of predictors of limb loss and mortality. Odds ratios with 95% confidence intervals (CI) were reported. *p* values lower than 0.05 were considered statistically significant.

## 3. Results

During the analyzed period, a total of 84 patients were referred for PVI of the lower limbs. Of them, 14 patients were excluded for the presence of an associated abdominal and/or thoracic injury and three more were excluded because the PVI was consequent to a gunshot. Finally, 67 patients were retained for the analysis. 

All patients were males. Their mean age was 30.1 ± 10.6 years old, ranging from 11 to 80 years old. Half of the patients (34 cases, 50%) had an age between 21 and 30 years old.

At arrival, most patients (57, 85.1%) presented with hard signs of vascular injury (Table 1), 66.7% of these cases being active bleeding. Concomitant signs of distal ischemia were present in 52 out of 57 patients (91.3%). The remaining ten patients had a non-pulsatile hematoma at the site of injury along with present distal pulses.

Twenty-one patients (31.3%) presented with hemorrhagic shock, and 17 more (25.4%) had a circulatory collapse. The mean systolic blood pressure at admission was 92 ± 25 mmHg, ranging from 40 to 150 mmHg, while the initial mean heart rate was 103 ± 21 bpm, ranging from 54 to 140 bpm.

Altogether, 50 patients (74.6%) arrived at our emergencies with a local hemostatic compression.

In laboratory tests, the mean hemoglobin level was 8.2 ± 2.5 g/dL, ranging from 4 to 14 g/dL. Forty-two patients (62.7%) needed a blood transfusion. 

The wound localization was mainly at the thigh (upper and medial in 42 patients, 62.7%; distal thigh in 10 patients, 14.9%), while 13 patients had a wound situated below the knee (19.4%). In the remaining two cases, the wound was retroarticular.

**Figure 1:**
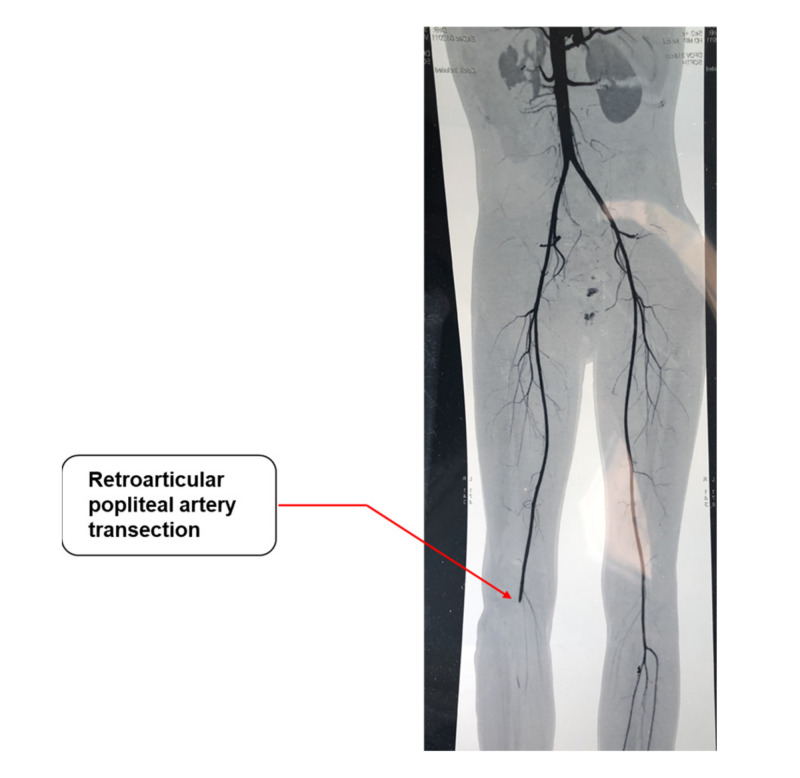
Angio CT showing a right retroarticular popliteal artery transection (red arrow).

In particular, 29 patients (43.3%) had an isolated arterial injury of the lower limbs and 6 patients (9%) had an isolated venous injury, while 32 patients (47.8%) had a combined arterial and venous injury. Seven patients (10.4%) also had an associated nerve injury, being in six cases a sciatic nerve injury (9%) and in the remaining patient an internal sciatic popliteal nerve injury. The arterial injuries mainly involved the superficial femoral artery (Table 2) and were in most cases either transection (31 cases, 46.2%) (Figure 1) or lacerations (21 cases, 31.3%). Furthermore, the most affected site in case of venous injury was the superficial vein (18 cases, 26.8%), and transections and lacerations were the most represented type of lesions (17 and 19 cases, respectively; Table 2).

On presentation, all patients were assessed, and resuscitation protocols were initiated if signs of hypovolemic shock were present. Then, the patient was referred to the operation room according to the clinical status. The average delay between the occurrence of the injury and the vascular repair was 6 h and 15 min, ranging between 1 h and 18 h. Proximal and distal control was obtained and Fogarty embolectomy with heparin flush performed on all patients prior to further repair where needed. In two cases, the embolectomy was sufficient alone (See Table 3).

Arterial repair was mainly performed using either vein graft bypass (in all cases, the contralateral reversed autogenous great saphenous vein being the graft of choice) or direct arteriorrhaphy (15 patients each, 22.4%; Table 3). In 12 cases, vascular ligation was performed without any possibility of reconstruction. In particular, in six cases the ligation involved collaterals of the deep femoral artery, while in the remaining six cases the ligation was required for one tibial artery.

Furthermore, different techniques for venous repair were performed: in most cases, vein patch angioplasty (20.9%), vein ligation (11.9%) and direct venorrhaphy (10.4%).

Fasciotomy was performed in two patients (3%) who presented with tense compartment syndrome. 

No primary amputations were performed. 

Patients’ average length of stay was 6.3 ± 5.4 days, ranging from 1 to 30 days.

### 3.1. Postoperative Complications

#### 3.1.1. Mortality

Two patients died in the postoperative course, representing 3% of the cases (Table 4). In particular, one patient presented in a severe hemorrhagic shock state for an above-knee popliteal artery transection. After resuscitation, the patient had a popliteo-popliteal bypass vein graft. The immediate postoperative course was marked by the restoration of the limb vitality, but the patient did not survive a severe metabolic acidosis with an acute renal failure, despite renal replacement and invasive ventilator support in the intensive care unit. Death was declared on the ninth postoperative day. 

Similarly, the second patient arrived with a severe hemorrhagic shock state for a common femoral artery transection. After resuscitation, the patient had a femoro-femoral vein graft bypass with reimplant of the deep femoral artery, but the patient did not survive to the occurrence of a disseminated intravascular coagulation.

#### 3.1.2. Amputation

Three patients (4.5%) had a lower limb amputation after failed revascularization (Table 4). In particular, one patient had a common femoral to superficial femoral bypass vein grafting for a superficial femoral artery transection, with an extremely deteriorated lower limb tissue. The bypass thrombosed on the third postoperative day, and any attempt to revascularization failed. Therefore, transfemoral amputation was performed. 

The other two patients had a popliteo-popliteal bypass vein grafting for a below-the- knee popliteal artery transection, along with skin state deterioration. The bypass thrombosed the second day following the operation, requiring urgent re-exploration, but the following day the graft reoccluded without any possibility to restore the flow to the limb. A transfemoral amputation was then performed.

#### 3.1.3. Other Complications

As described in Table 4, the most frequent postoperative complication was represented by an operative site infection in five patients (7.5%) who required reintervention and were treated with a surgical drainage (four patients) or the placement of an irrigation aspiration system in the remaining patient; the other four (6%) had a surgical drainage. For all these patients, an intravenous antibiotico-therapy was initially administrated and later adapted based on antibiogram.

### 3.2. Predictive Factors Associated with Limb Loss and Mortality

In the univariate analysis, no factor was found to be significantly associated with either limb loss or mortality. However, when the outcomes were considered together, several factors were found to be predictive of the combined event of mortality and limb amputation (Table 5). 

In particular, among the preoperative clinical factors, the presentation with either hemorrhagic or compartment syndrome, the presence of nerve paralysis and the absence of external pre-hospital hemostatic compression all significantly increased the risk of postoperative mortality and limb loss. 

The site of arterial injury also represented a significant predictor of amputation and mortality, particularly if the lesion was located at the superficial femoral artery (OR 4.32, *p* = 0.001) or at the popliteal artery (OR 4.89, *p* = 0.0015). The type of lesion also affected the risk of mortality and limb loss, the arterial transection being significantly predictive (*p* < 0.0001). Furthermore, the presence of a combined arterial and venous injury increased by 3.82 times the odds of limb loss and mortality (*p* = 0.023, 95%CI: 1.86–4.04). 

From the different surgical arterial repair techniques, the vein graft bypass was the only technique associated with a significant odds of limb loss and mortality (*p* < 0.0001).

The length of in-hospital stay was also a significant predictor of limb loss and mortality (*p* = 0.004), the risk being higher the longer the stay.

In the multivariate analysis, the need for a vein graft bypass remained the only significant predictive factor associated with limb loss and mortality (*p* < 0.0001), with an odds ratio of 4.58 (CI 95% 1.44–6.03).

## 4. Discussion

Penetrating vascular injuries to the lower limbs due to stab wounds are becoming more frequent among civilians in urban areas [8] and may cause a variety of complex injuries depending on the site and type of lesion to the vessels and the surrounding tissues. These injuries have a potential to cause high rates of mortality and limb loss, especially in a young population, as a result of hypovolemia, ischemic syndrome or wound infections if they are not timely recognized and properly treated.

In the literature, the reported mortality rates range from 1.6% [1] to 8.5% [9].

Therefore, it is crucial to identify the most important issues that can be relevant to optimizing practice management with the aim to ameliorate the outcomes and reduce rates of mortality and limb loss.

According to our results, the clinical presentation of the patient plays a key role, and therefore the evaluation of the injured extremity after penetrating trauma is of utmost importance [10]. 

There are two main approaches when evaluating patients presenting with PVI of the lower limbs. The first approach is based on the presence of hemorrhagic syndrome and/or ischemic syndrome, while the second approach focuses on the presence of hard or soft signs of vascular injury. 

Nevertheless, the presence of hemorrhage and ischemic syndrome are both considered hard signs of PVI; therefore, the second approach is the most used to determine the need for immediate surgery [11] or the possibility for further imaging investigations in case soft signs are present [10].

According to most authors, the absence of hard signs on physical examination essentially excludes the presence of clinically significant arterial injury. Dennis and colleagues [12] evaluated 287 patients with penetrating lower extremity trauma who did not have hard signs of vascular injury. They observed only four patients (1.3%) who had delayed onset of hard signs and ultimately required surgical repair, while the remaining 283 patients were discharged after 24 h without any significant complication.

In our study from a total of 67 patients, the majority presented with hard signs of vascular injury. 

Dividing patients based on the presence of hard and soft signs of vascular injury is an easy and efficient way that facilitates the sorting of the patients at admission and therefore clarifies the further management, enabling to save crucial time when dealing with these patients.

However, before attending the hospital, the local hemostatic compression achieved by manual compression and pressure bandages is of utmost importance as it could be a life-saving gesture [10], given the fact that often patients arrive with hemorrhagic syndrome. In our experience, the absence of local hemostatic compression on admission and the presence of hemorrhagic syndrome on arrival were both strong predictors of the risk of mortality and limb loss. 

Moreover, the presence of nerve paralysis and/or compartment syndrome significantly raised the risk of mortality and limb loss (about four times each), both being typical signs of advanced ischemic damage. Nevertheless, the presence of a local neurologic compression due to an associate nerve injury was not a predictor of limb loss and mortality. 

These results were in line with those reported by Hafez and colleagues [1], who identified the presence of tense compartment and/or limb neurologic deficit at presentation as the main clinical predictive factors of amputation and mortality. 

In addition, Perkins and colleagues [2] in their meta-analysis reported that prolonged ischemia and the development of compartment syndrome were associated with a fourfold and fivefold increase in the risk of secondary amputation, respectively. On the other hand, the presence of hemorrhagic shock on admission was not associated with a significant increase in secondary amputation. 

The site of vascular injury was another important prognostic factor in our series.

Based on the literature, the arteries most commonly involved in penetrating traumas are the superficial femoral artery (SFA) and the popliteal artery. In the series of 59 patients reported by Kruger and colleagues [8], the PVIs involved the SFA in 57.6% of cases and the popliteal artery in the remaining patients. Kauvar and colleagues [13] found that more than half of their 431 patients had an arterial injury either in the SFA (27.8%) or in the popliteal artery (35.5%). Our results were consistent with those reported in the literature. Furthermore, according to our results, the location of the injury at the SFA or at the popliteal artery was significantly associated with the risk of mortality and limb loss, with an odds ratio of 4.32 and 4.89, respectively. In a similar fashion, Mirdamadi and colleagues in their study of 112 patients found that those who had injuries to femoral and popliteal regions, among those who experienced lower limbs trauma, were likely to face worse outcomes [14]. Similarly, Hohenberger and colleagues found poorer functional outcome after vascular extremity trauma to the lower limbs than to the upper limbs in their retrospective experience of 27 patients treated for arterial injuries [15].

In addition, the type of arterial injury, and in particular the presence of arterial transection, was a significant predictor of limb loss and mortality in our experience. Arterial transection, which was the most frequent type of lesion reported in our case series, was also correlated to the worst clinical presentation, which was hemorrhagic syndrome with associated ischemic syndrome, and all patients who presented with transection had an active bleeding. 

When a PVI is present, the surgical arterial management has two main goals: bleeding control and arterial repair. 

In our study, arterial repair was achieved using a vein graft bypass in many cases. This is explained by the fact that penetrating injuries of the lower limbs are associated with large lesions, and vein grafts are preferable to prosthetic grafts due to a high risk of local infections. Furthermore, according to Perkins and colleagues [2], injuries repaired with a prosthetic interposition graft had a higher risk of amputation and mortality than those repaired with an autologous vein. 

Nevertheless, in our study, the need for a vein bypass graft was the only predictor of limb loss and mortality in the multivariate analysis. Again, this issue could be explained by a worse systemic and local presentation of patients requiring this procedure compared to patients requiring direct arterial repair or end-to-end anastomosis. 

Nevertheless, recent literature favors the use of contralateral or even ipsilateral great saphenous veins for bypass and as durable conduits in cases of lower extremity arterial trauma [16].

A very limited role for primary amputation exists in the management of penetrating extremity vascular injuries. As the majority of the injured patients are young, repair should always be prioritized, and primary amputation should only be considered in patients with extensive soft tissue and neurovascular disruption who have life-threatening associated injuries. Delayed amputation after a vascular repair is the nightmare of every vascular surgeon as it signals the failure of the revascularization. Most of these amputations are related to graft occlusion, but some cases of extensive infection requiring amputation were described in the literature [17,18]. Intraoperative or in-hospital occlusion of an arterial repair is usually related to delayed presentation of the patient after injury, delayed diagnosis of the injury by the physician, a technical issue in the operating room, or occlusion of venous outflow from the area of injury. In particular, technical issues during the operation that may lead to postoperative thrombosis of a repair include too much tension on an end-to-end anastomosis, failure to remove any thrombi or emboli in the distal arterial tree with a Fogarty embolectomy catheter, narrowing of a circumferential suture line, and failure to flush the proximal and distal arteries before final closure of the repair. 

In our case study, the rate of secondary amputation after failed revascularization was 4.5%, which was in line with the 4% rate reported in the study of Kruger and colleagues [8], and with the 5% rate reported by Perkins and colleagues [2]. However, Liang and colleagues [19] reported the highest secondary amputation rate of 17%.

In the literature, to the best of our knowledge, this is the first study on PVI of lower limbs specifically due to stab wounds, which makes the related predictive factors studied more precise and targeted.

However, this study has some limitations, including the small sample size, the retrospective design and the lack of long-term follow-up, which prevent the generalization of the results.

## 5. Conclusions

PVIs of lower limbs due to stab wounds are serious injuries that were lethal in 3% of cases and lead to a secondary major amputation in 4.5% more cases. The clinical presentation (hemorrhagic syndrome or compartment syndrome, presence of neurologic paralysis, absence of external pre-hospital hemostatic compression), localization of the arterial injury (superficial femoral artery or popliteal artery), presence of arterial transection and the need for a vein bypass grafting were all significant predictors of limb loss and mortality in the univariate analysis. In the multivariate analysis, the need for a vein bypass grafting remained the strongest predictor of limb loss and mortality at 30 days postoperatively.

## Figures and Tables

**Table 1:** Distribution of patients presenting with hard signs of vascular injury.

Hard Signs of Vascular Injuries (*n* = 57)	Number	Percentage
Active bleeding	38	66.7%
Expanding hematoma	7	12.3%
Pulsatile hematoma	11	19.3%
Thrill or bruit	1	1.7%
Concomitant distal ischemia	52	91.3%

**Table 2:** Details of the localization and the type of injury for arterial and venous lesions.

Site of Vascular Injury	Location	Type of Injury
Arterial: *n* = 61 (91%)	Common femoral: 4	Transection: 31 Laceration: 21 False Aneurysm: 5 AVF: 1 Spasm: 2 False aneurysm + AVF: 1
	Superficial femoral: 23	
	Deep femoral: 10	
	AK popliteal: 8	
	Retrogenicular: 2	
	BK popliteal: 4	
	Anterior tibial: 4	
	Posterior tibial: 5	
	Superficial + deep femoral: 1	
Venous: *n* = 38 (56.7%)	Superficial femoral: 18	Transection: 17 Laceration: 19 Fistula: 2
	Deep femoral: 4	
	AK popliteal: 6	
	BK popliteal: 3	
	Posterior tibial: 1	
	Anterior tibial: 1	
	Great saphenous vein: 3	
	Common femoral + Deep femoral: 1	
	Superficial femoral + deep femoral: 1	

AK = above the knee; BK = below the knee; AVF = arterial-venous fistula.

**Table 3:** Details of the different arterial surgical repair techniques used in the presented case series.

Techniques	Number	%
Vein graft bypass	15	22.4
Vein graft interposition	4	6
Prosthetic graft	1	1.5
Direct end to end anastomosis	5	7.5
Vein patch angioplasty	3	4.5
Lateral arteriorrhaphy	15	22.4
Pseudoaneurysm open repair + vein graft interposition	2	3
Ligation	12	16.4
Fistula disconnection	2	3
Fogarty Embolectomy	2	3

**Table 4:** Details of postoperative complications.

Postoperative Complications	Number	Percentage
Death	2	3%
Amputation	3	4.5%
Wound infection	5	7.5%
Vein graft bypass thrombosis	4	6%
Bleeding	1	1.5%
Arterial thrombosis	1	1.5%
Pneumopathy	1	1.5%

**Table 5:** Univariate analysis of different factors related to amputations and mortality after penetrating vascular injuries of the lower limbs.

Risk Factor	Amputation and Mortality		
	Odds Ratio	95% CI	*p* Value
Age	1.54	0.48–4.96	0.539
Hemostatic compression	3.92	1.98–5.89	0.0001
Delay between arrival and treatment	1.28	0.39–2.26	0.24
Delay between injury and treatment	1.02	0.35–1.96	0.15
Paralysis	3.86	1.9–8.85	0.002
Compartment syndrome	4.12	1.98–5.01	0.006
Hemorrhagic syndrome	4.39	1.72–6.33	0.006
Location of the injury at SFA	4.32	1.66–5.44	0.001
Location of the injury at popliteal artery	4.89	1.86–5.92	0.0015
Arterial transection	4.82	1.98–5.98	<0.0001
Combined arterial and venous injuries	3.82	1.86–4.04	0.023
Associated nerve injury	1.34	0.77–1.98	0.896
Vein graft bypass	2.94	1.05–5.03	<0.0001
Length of stay	4.91	2.3–6.08	0.004

CI = confidence interval; SFA = superficial femoral artery.

## Data Availability

Data will be available upon request to first author.
